# Water–Energy–Food Nexus in the Agri-Food Sector: Research Trends and Innovating Practices

**DOI:** 10.3390/ijerph182412966

**Published:** 2021-12-08

**Authors:** Víctor Correa-Porcel, Laura Piedra-Muñoz, Emilio Galdeano-Gómez

**Affiliations:** Department of Economics and Business, University of Almería (Mediterranean Research Center on Economics and Sustainable Development, CIMEDES, Agrifood Campus of International Excellence, ceiA3), 04120 Almería, Spain; victorcorreaporcel@hotmail.com (V.C.-P.); galdeano@ual.es (E.G.-G.)

**Keywords:** Water–Energy–Food Nexus, agri-food sector, bibliometric analysis, innovation practices, sustainability

## Abstract

Natural resources are becoming scarcer and, together with the growth of the population, a widespread situation of overexploitation is inevitable that has become the biggest challenge for today’s world. In this context, the agri-food sector has a considerable environmental impact in terms of water and energy consumption. For about two decades, the Water–Energy–Food Nexus (WEF) Nexus has been trying to address this problem, focusing on efficient interrelationships among these dimensions. The objective of this work is to analyse the evolution of research on WEF Nexus in the agri-food sector and its development in scientific databases. For that purpose, a bibliometric study was carried out with publications obtained from the Scopus database, examining the main journals, authors, institutions, countries, subject areas, funding sponsors, and keywords. Moreover, a final section is specifically dedicated to the agri-food innovations in WEF Nexus in order to explore innovative aspects to effectively overcome technical barriers that hinder a real implementation of the Nexus approach. The results show that, over the past decade, Nexus research in the agri-food sector has been growing exponentially. The top country in this field is USA, the most studied area is environmental science, and the most relevant keywords are “energy use”, “water budget”, “food security”, “sustainable development”, and “water resources”.

## 1. Introduction

Following past failures in Water–Energy–Food (WEF) resource management, academics, policy makers, and planners proposed a nexus approach to understand the synergies, trade-offs, and spill-over effects of interconnecting these components [1]. The origins of the WEF Nexus go back to the United Nations Scientific Conference on the Conservation and Utilisation of Resources (1949), where more than 600 scientists from some 50 countries discussed the sustainable management of the needs of a growing population [2,3]. This has been followed by many workshops, projects, and conferences focused on the study of Nexus components [4]. With the food crises in developing countries in 2008, concerns about the food–energy nexus became even more important [5]. Thus, the WEF Nexus has become popular since the World Economic Forum Annual Meeting 2008, held in Davos (Switzerland). The main leaders in business world agreed to call water “the link between economic growth and environment” [6]. The global goals related to economic development from the Nexus position were recognised. Already in 2015, the United Nations introduced the Sustainable Development Goals (SDGs), a marriage of science and policy [7,8] with the intention to be implemented globally by 2030 [9]. The SDGs directly linked to the Nexus would be SDG 2 (food security), SDG 6 (clean water), and SDG 7 (modern energy), and indirectly SDG 13 (climate change) and SDG 15 (terrestrial ecosystems) [10].

Water is an indispensable element in the food industry. Specifically, the activity that consumes 90% of the fresh water in the world is agriculture [11], also using about 25% of the global energy in the production and supply of food [12]. Among the different types of agriculture, the predominant one is irrigated agriculture, occupying one-fifth of the world’s cropland and providing about 40% of global food production, with an estimated 280 million hectares of cropland [13]. Thus, the importance of irrigated agriculture has been reinforced due to the growing demand for food, the increasing world population and the trend to use more and more organic products. In water-scarce regions, it becomes essential to maintain agricultural production, especially in environments undergoing transformation processes, as it is a vital source of income in rural areas with fewer resources [14,15]. Despite this, in regions that are prone to drought, water use for irrigation is an impediment to water use in agriculture and energy production [16]. Water abstraction has increased dramatically in recent decades, due to the expansion of cropland, improved irrigation technology, and political incentives, which could result in many of the world’s freshwater bodies being unable to sustain agricultural production in the foreseeable future [17]. Consequently, the Nexus should be deeply analysed in this sector, as a common framework that is able to bring together and link these three indispensable elements for human development, while managing them efficiently.

For this reason, this article focus in the agri-food sector and the objectives are (I) to give the reader a historical overview of the origin of the Nexus, (II) to undertake a bibliometric analysis of the WEF Nexus in the international agri-food sector, (III) to highlight the main innovations of the Nexus in this industry, and (IV) to make a constructive criticism of the prospects of the Nexus for future research.

The usefulness of bibliometrics is due to the abundant amount of scientific information produced in recent years, associated in turn with new forms of communication, which has led the research community to propose its measurement. A bibliometric analysis uses mathematical and statistical methods that make it possible to obtain reliable indicators associated with scientific activity about a specific topic. It permits to explore large volumes of scientific data. In this way, it is possible to obtain information about the number of documents published by a country or institution, the research groups, or individuals with the highest scientific productivity. This allows us to identify research trends and gaps in this topic, promoting scientific discussion.

Thus, the main WEF agri-food studies are analysed through the information obtained from the Scopus database, examining the main journals, authors, institutions, countries, subject areas, funding sponsors, and keywords. Additionally, a final section is specifically dedicated to the agri-food innovations in WEF Nexus. In the review, we have found mainly theoretical but less empirical research involving innovating practices. Nevertheless, one of the main challenges affecting the Nexus operationality is developing and evaluating innovative solutions to effectively overcome social, economic, or technical barriers that hinder a real implementation of the Nexus approach. For this reason, it has been considered very relevant to dedicate a specific section to innovating practices in WEF Nexus in order to find technical solutions and to identify innovative strategies to improve the Nexus implementation.

For the purposes of this research, the present study is structured as follows: Section 2 shows the literature review; then, Section 3 explains the sources from which the information provided has been extracted and the various computer tools used to structure this information; Section 4 contains the results, i.e., the bibliometric analysis itself, grouped into different study categories: main journals, most prolific authors, institutions most involved in the study of the WEF Nexus in the agri-food sector, and the main keywords; Section 5 identifies the most relevant innovations and their implications for the efficiency of the WEF Nexus. Finally, Section 6 provides the main conclusions.

## 2. Theorical Framework

Since its origin in 2008, there have been multiple interpretations of Nexus [4,18,19], covering from more systemic perspectives [20,21] to more sociological ones [22]. This lack of unification makes the Nexus inconsistent, rather than serving as a basis for linking ideas and achieving environmental sustainability. Geopolitical differences that cause the Nexus to be defined in different ways must be set aside [23], avoiding creating a multidisciplinary term based exclusively on regional characteristics [24,25]. In addition, the sheer complexity of the Nexus and the emerging technology mean that there is ample reason to believe that there are opportunities for future research, particularly in the field of agricultural economics and sustainable resource management. The difficulty in the interactions of the WEF Nexus makes it necessary to employ systemic approaches in order to comprehensively assess policies [26]. At the same time, collaboration between researchers from different disciplines, policy makers, and agricultural practitioners [27,28] is needed to avoid cases of uncoordinated approaches such as crop insurance, which hinder innovation and adoption of energy crops. Data availability thus becomes critical for the development of the Nexus [29,30]. Although machine learning has proven useful for prediction, efficient policy design through causal relationships is usually necessary [31], making the handling of big data from emerging agricultural technologies a line for future research.

Nexus has been increasingly developed during recent years, so both the public and private sectors have funded many research projects related to it [32]. Most authors dealing with the WEF Nexus topic focus their research mainly on two issues: the social dimensions of the Nexus (cultural, political, etc.), and quantifying its interrelationships. Nevertheless, there was not a clear consensus about the Nexus definition when it originated in 2008 [33]. In place, varying interpretations in different questions were developed, trying to be grouped in two categories of definition.

The first one defines Nexus as the interaction of different subsystems [34], i.e., the interdependences among energy and water; they are coupled in a procedure with the following steps: supply, processing, distribution, and use [33]. Equally, when we extend the limits to the water–energy–food system, the Nexus can be defined as the connexion between water, energy, and food [20]. Therefore, water is essential to produce energy and food. Energy is required for the process of water treatment and food can be used to produce biofuels (energy). In short, this category is focused on showing relations between different subsystems, with the purpose to grasp the general features of this complex system by its component’s linkages [33]. A practical example would be the case of Latin America and the Caribbean (LAC, see Abbreviations) [35]. Although the Nexus studies conducted in this region of the planet are relatively scarce and have only addressed partial interrelationships between the parts of the WEF Nexus, they have provided an analysis focused on water and food security based on the progress made at the social and environmental levels [36]. They highlight the importance of agriculture in LAC for global food security and water quality and water footprint, and the impact on biodiversity and carbon stocks [37,38]. Leaving aside the water-=–food study, the water–energy study has considered the water balance and water footprint implications of unconventional oil and gas extraction. Water and energy are vital resources for the food system [39], and their assessment can help minimise environmental emissions [33], as well as becoming a key sub-component in the broader efficiency goal of the Water–Energy–Food Nexus. However, LAC countries have not incorporated Nexus thinking into their policies, which is critical for sustainable development in this part of the world [40,41].

The second category, and the most followed since then, presents the Nexus as an analysis focus on quantifying these connections. First, the FAO (Food and Agriculture Organization of the United Nations) pointed out that the functions of this Nexus approach were to analyse the binomial human-nature, and to generate an integrated administration of natural resources through different scales and sectors by constructing managing trade-offs and synergies [42]. One example is the introduction of agroforestry in Africa. Agroforestry is a production system that integrates trees, livestock, and pasture in the same production unit, with the aim of improving land productivity and, at the same time, being ecologically sustainable [43]. Thus, in the Sahel, trees are commonly used as windbreaks, feed for livestock, as well as for fuelwood and food production [44]. Indirectly, the benefits of agroforestry have led to poverty reduction strategies, such as mitigating school dropout rates [45], and it has even been promoted in the international Reducing Emissions from Deforestation and Forest Degradation (REDD+) programme, which aims to reduce carbon emissions by improving the livelihoods of African farmers [46]. On the other hand, in South America, overexploitation of water for unconventional oil and gas extraction competes with other water uses [47]. Research efforts in this region should be done at the regional level to provide a solution to water scarcity in vulnerable watersheds, but due to the heterogeneity of the LAC area, little attention is paid to the needs of communities, households, and small businesses that suffer from this problem [48]. Finally, Nepal, which despite being one of the countries with the richest water resources [49], has poor and non-inclusive access to drinking water and a system vulnerable to contamination [50].

Thirdly, WEF Nexus can also be analysed from a sociological point of view. According to Voelker et al. [22], the WEF Nexus is a link between the following aspects: social changes related to behaviours towards environmental care, important enough to involve legal reforms; and the delimitation of the Nexus, measurement systems and indications issued in this area by the European Commission. Thus, such is the importance of environmental sustainability that many authors agree to call the Nexus as Water–Energy–Food–Ecosystems (WEFE) Nexus [51,52]. Returning to the topic of agroforestry, Torralba et al. [53] conducted an analysis in Europe in which they observed an improvement in biodiversity and soil fertility. Subsequently, Brandt et al. [54] observed that in some areas of the Sahel, farmers are very interested in organic products such as fuelwood, due to the income obtainable from selling them, to the point of promoting tree cover around villages. Finally, following the outbreak of the War of Darfur (Sudan) in 2003, many refugee camps have been built around agricultural markets, converting high-quality farmland into wasteland [55].

Following Scott et al. [56], the gist of Nexus is to generate a recovery of resources, using the subproduct of resource usage efficiency upgrades. Meanwhile, Smajgl et al. [18] said that the Nexus was an ever-evolving system, citing that the interactions between Nexus nodes should be dynamically tackled. The Nexus is so rich for Keskinen et al. [19] that they argued it could not be interpreted from a single perspective but three, which complement each other: an analytical method, a governance tool, and an emerging discipline.

Despite the large amount of literature about the Nexus, there is still a debate about how best to transform it from a theoretical system to real policies [12]. Shannak et al. [57] pointed out a number of key factors that the authorities should be mindful of, such as the need for a methodical and quantitative assessment of the energy that is consumed, a level of country and both for water and food, which rely on refined models to assess the Nexus across sectors and actors. On the other hand, Zhang et al. [33] proposes the creation of a socio-economic model that is capable to predict, in the different periods, the needs of inputs, represented in socio-economic demands, productive costs, and limitations in environmental care. Additionally, Terrapon-Pfaff et al. [48] suggest, for a local level, a simplified four-step evaluation system.

In sum, the Nexus purpose is to create an integrated administration of the three dimensions (water, energy, and food) by their mutual coordination to reduce unexpected trade-offs and stimulate a sustainable development [58,59]. It is precisely the lack of coordination between dimensions the main barrier to the effective implementation of the WEF Nexus [58].

## 3. Sources and Tools

A bibliometric analysis has been applied in order to analyse the evolution of research on WEF Nexus in the agri-food sector [60]. Bibliometrics applies mathematical and statistical methods to all scientific literature and the authors who produce it, with the aim of studying and analysing scientific activity. It has been frequently used for the systematic evaluation of scientific publications and the identification of research phenomena in a particular field [61].

**Figure 1:**
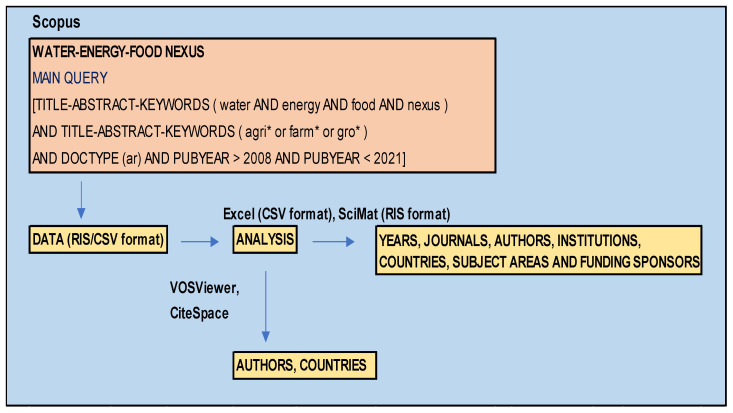
General scheme of research methodology. Source: own elaboration.

Publications were obtained from the Scopus database, considered internationally as one of the most complete bases in the field of scientific research [62], using search parameters related to terms of water, energy, food, and agriculture (Figure 1). This keywords’ selection was made based on similar studies about WEF Nexus and bibliometrics [61,62]. We only used the “article” format, with the objective to avoid information duplication [63]. The study period starts in 2008, as it is when the first agri-food WEFE paper is found. The search was developed in 2021 and the final sample analysed was composed of 463 articles.

Once the sample was selected, we processed the data using various tools. First, we filtered the information to avoid duplications, mistakes, and omissions. Then, different variables were analysed: main journals, authors (and their institutions and countries), subject areas, funding sponsors (private and public), and keywords.

The selection criteria for these variables are based on the study of a sample of the most prolific components of each of them. These are the most common variables that are analysed in bibliometric studies when the aim is to study the trends of a given topic [1,9,60]. Firstly, we filtered according to the number of articles to obtain the 10 most important components within the journals, institutions, countries, and funding sponsors variables; secondly, we used average citations for authors; and finally, we opted for a graphical representation method for study areas and keywords. For the final section on innovations in the WEF Nexus, a less static criterion was used, first ordering the publications according to the number of citations and then choosing those that most closely resembled our focus of analysis within the WEF Nexus: the agri-food sector.

The tools used to process the data are Excel (2016 version; Microsoft, Redmond, DC, USA), SciMAT (1.1.04 version; Soft Computing and Intelligent Information Systems Research group; University of Granada, Granada, Spain), VOSviewer (1.6.14 version; Leiden University, Leiden, The Netherlands), and CiteSpace (5.0.R2 SE version; University of Drexel, Philadelphia, PA, USA).

## 4. Results

### 4.1. Evolution on the Main Parameters of Water–Energy–Food Nexus (WEF Nexus)

**Figure 2:**
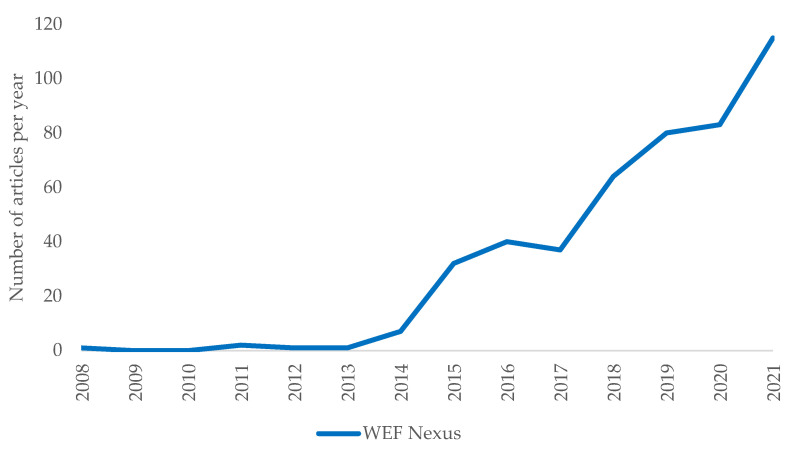
Annual evolution of the number of articles. Source: own elaboration.

Table 1 shows the evolution of the principal variables related to agri-food WEF Nexus studies. The first records date back to 2008, which means that the WEF Nexus is a recent research area. During the last 13 years, the number of articles has grown from 1 in 2008 to 115 in 2020 (Figure 2). Around 75% of the articles were published after 2018, when a large increase in the number of publications took place.

### 4.2. Main Journals

The total number of journals about WEF Nexus topic in the agri-food sector is 160. To facilitate the study of the Nexus, we selected the top 10, as shown in Table 2. The total number of articles published by the selected journals sample is 176, representing 38% of the total.

Journal of Cleaner Production is the most productive journal with 30 published articles, which is almost 7% of the sample. It began publishing about the WEF Nexus in 2015, making it one of the newest journals in this ranking. It has an H Index of 13 (200 in the global). Science of the Total Environment ranks second according to the number of articles, with 27. It has the highest H Index, both at the sample level (15) and at the general level among these journals (244). It is also the most years-long journal, publishing since 1972. Water (Switzerland) is third in this list, with 24 articles. Despite this, is has a sample-level H Index of 6, one of the lowest among the 10 journals, being 55 at the journal level. It also has the lowest average citations (8.13 citations per article). These low figures may be due to the time that it is one of the most recent journals of founding (2016). On the other hand, Applied Energy has the highest score in SJR, with 3.035 (Q1), and the journal with the highest average citations (37.5 citations per article). Netherlands is the country with the highest number of publications.

### 4.3. Most Relevant Authors

To choose the most prominent authors in this topic, we first selected the 10 authors with the most publications and then ordered them according to the highest number of average citations (Table 3). This is because the order of published articles does not provide any relevant information and, at the same time, Scopus does not allow direct ordering according to the number of average citations.

Paolo D’Odorico is in the first position with 52.2 citations per article. He has 261 citations in total and 5 published articles; he is also in the ranking author with the highest number of citations. His H Index is 5. D’Odorico has many studies on gas and oil extraction [47,64,65,66], but his most prominent work is one about the relation Nexus-biofuels [67].

The second place is for Qiang Fu, from Northeast Agricultural University (China). He has an average of 41 citations per article, with 164 citations and 4 articles. In these 4 articles, he shares authorship with the other two Chinese authors in this ranking: Mo W. Li and Dong Liu. Thus, all three work for the Northeast Agricultural University. Their works have been focused on the study of resource optimisation from the Nexus perspective [68,69].

The third position in this ranking is for Pietro Elio Campana (Mälardalens högskola, Sweden). He has an average number of citations of 36.25 (145 citations between the 4 articles published about the WEF Nexus). His H Index is 4 and he started publishing about the Nexus in 2015, making him one of the most veteran authors in this ranking. His research has focused on solving the problems caused by drought in agriculture [70,71].

From a more general point of view, there are two other authors with a closer longer relationship: Tareq Al-Ansari and Rajesh Govindan. The two work in Qatar (Hamad Bin Khalifa University). Al-Ansari started writing about the Nexus five years before Govindan, in 2015, and has a higher average citation rate (14.42 compared to Govindan’s 7.2). Their latest contributions have dealt with the implementation of the WEF Nexus in Qatar, both at a general level to guide the country’s national priorities [72], and at a more particular level in groundwater management [73].

**Figure 3:**
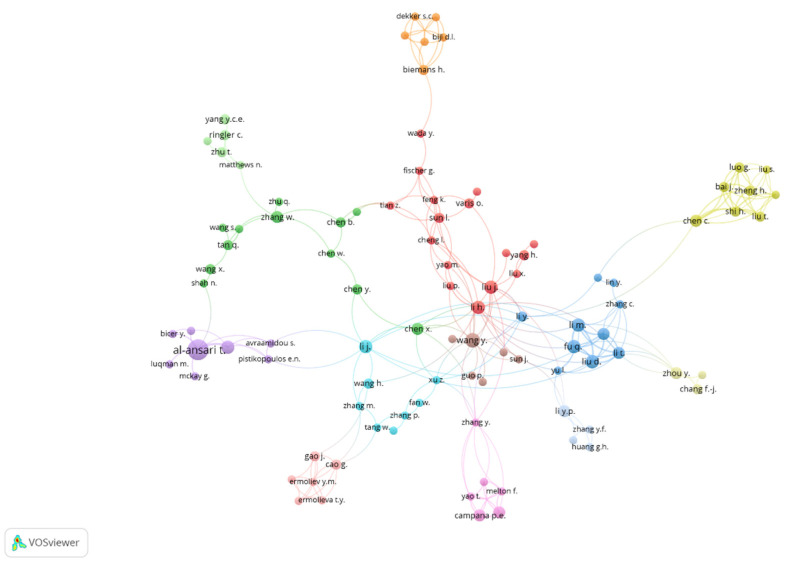
Co-authorship relations between WEF Nexus authors. Source: own elaboration.

With the objective of delving deeper into the relations between authors, Figure 3 graphically shows these links. Authors with at least two publications were selected. On the map, it can be seen that the most prolific authors of the WEF Nexus are distributed in clusters of different colours. Thus, in the blue cluster, we can see that Qiang Fu, Dong Liu, and Mo W. Li share authorship in five articles published about the WEF Nexus. Within the purple group, we can identify Tareq Al-Ansari and Rajes Govindan, with also five articles in common. At the bottom, we can find Pietro Elio Campana (pink cluster), although without any relation to other authors in the ranking in Table 3.

### 4.4. Most Productive Institutions

The 10 most relevant institutions in WEF Nexus were analysed (Table 4). Firstly, these universities accumulate around 25% of the articles of the sample, and most of them are in China. In fact, Texas A&M University is the institution with the highest number of publications with 17. It is followed by Beijing Normal University with 15 articles and Chinese Academy of Sciences with 14.

The institution with the highest average citations is The Royal Institute of Technology KTH, with 26.78 citations per article; it was founded in 1827 in Sweden, making it also the oldest institution in this ranking. At the country level, only China represents 30% of the total of the nations in our ranking.

### 4.5. Most Active Countries

The number of articles published in each country were considered, because here it gives a clearer picture of the relative importance of each nation (Table 5). The United States ranks first with 178 articles, more than doubling China, which ranks second with 78. The third place is for UK, with 59 articles. Only these three countries represent almost 70% of the articles in our sample. On the other hand, to make a more in-depth analysis, we will also talk about the average per capita citations, which is the number of citations per million inhabitants of each country. The podium would be here for Netherlands (1.5482 citations per million inhabitants), UK (0.8777), and Italy (0.5709). UK has the highest number of mean citations (22.24 citations per article). Finally, the country with the highest score on the H Index is USA with 30.

**Figure 4:**
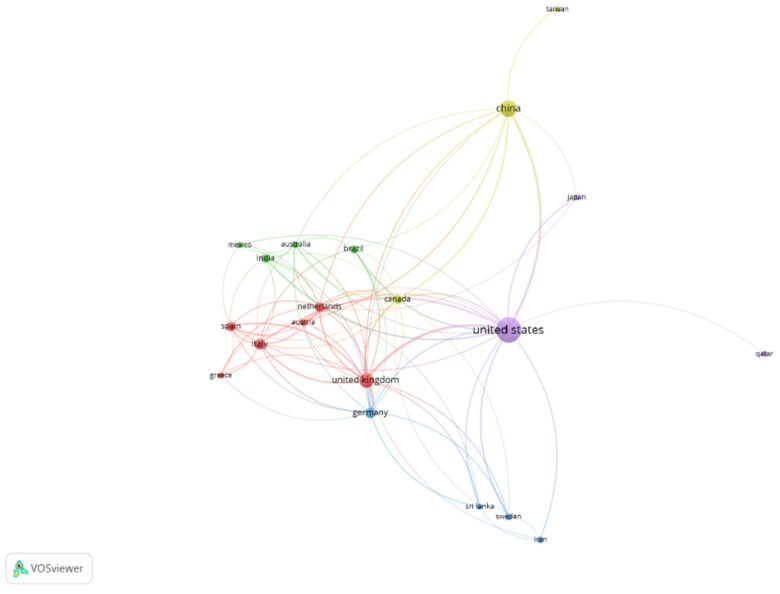
Relationships between WEF Nexus countries. Source: own elaboration.

In Figure 4, the relationship between the different countries can be observed. The clusters group those nations with a minimum of 10 published articles on this topic. Thus, in the resulting map, the size of the nodes will depend on the number of articles published by each country; the lines define the links between countries, and their thickness will be greater or lesser depending on the collaboration between them; and the coloured clusters indicate the main groups of collaboration. Several groups of countries can be distinguished. The first one is the red cluster, led by the UK, which also includes Italy, the Netherlands, and Spain. In the green cluster, we would find India and Brazil. China and Canada are part of the yellow cluster. USA (purple cluster) and Germany (blue cluster) would be separated from the rest of the countries in this ranking in two different clusters.

### 4.6. Principal Subject Areas in the Study of WEF Nexus

**Figure 5:**
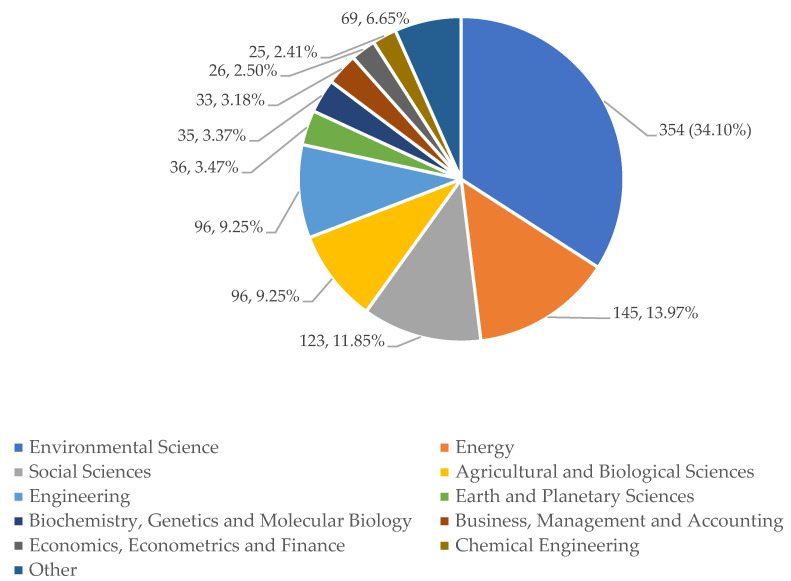
Distribution of the number of articles by WEF Nexus study areas. Source: own elaboration.

There are 21 subject areas to classify the WEF Nexus studies of our sample. Each article can be inside more than one of these categories. Subject areas are the different disciplines that show the main themes of an investigation. As we can see in Figure 5, the main subject area is Environmental Science with a percentage of 34.1, followed by a great distance by Energy (14%). Only these two already represent almost half of the themes of the articles. Social Sciences is near Energy (11.8%).

As observations, the main subject areas of the WEF Nexus are directly related to Sustainability and the Environment. As pointed out above, Social Sciences also have prominence. Other branches of Applied Science and Engineering have a much smaller role. Finally, Economy and Finance, and Pure Sciences, occupy a position almost irrelevant, drawing as a conclusion that the WEF Nexus focuses preferably on the study of the ecosystem, leaving aside economic efficiency or profitability, that is, the cost–benefit ratio, and strictly theoretical studies.

### 4.7. Most Important Funding Sponsors

The funding sponsors are the organisations or programmes responsible for financing researchers so that they can conduct their studies. In our case, many of them are linked to environmental and sustainability issues, related to the WEF Nexus (see Table 6). Thus, we will start analysing these systems according to the number of published articles. National Science Foundation (USA) is in the first position with 50 articles, and it has the highest H Index too (16). The National Natural Science Foundation of China is close behind, with 49 articles. Third place would go to the European Commission, but at a great distance from the above, with only 15 articles published about the WEF Nexus.

By country, there are three funding sponsors from China, representing one third of this ranking. It is followed by the European Union (EU) and Brazil, with two institutions or programmes each. The USA, UK, and France only have one funding sponsor.

The most ancient funding sponsor is the Chinese Academy of Sciences. It was founded in 1949. On the other side, the newest institution is the National Key Research and Development Program of China (2016), from China too. All these data only reinforce the current superiority of this Asian country in the WEF Nexus research.

### 4.8. Keywords: Relationships and Analysis

**Figure 6:**
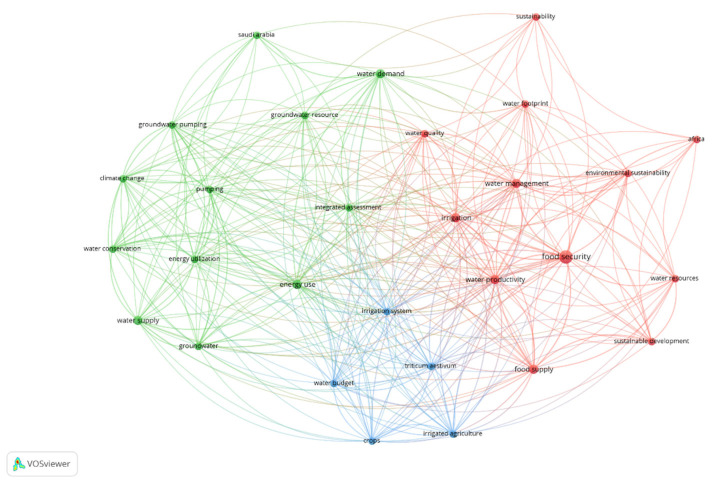
Most frequent topic words in the period 2008–2014. Source: own elaboration.

**Figure 7:**
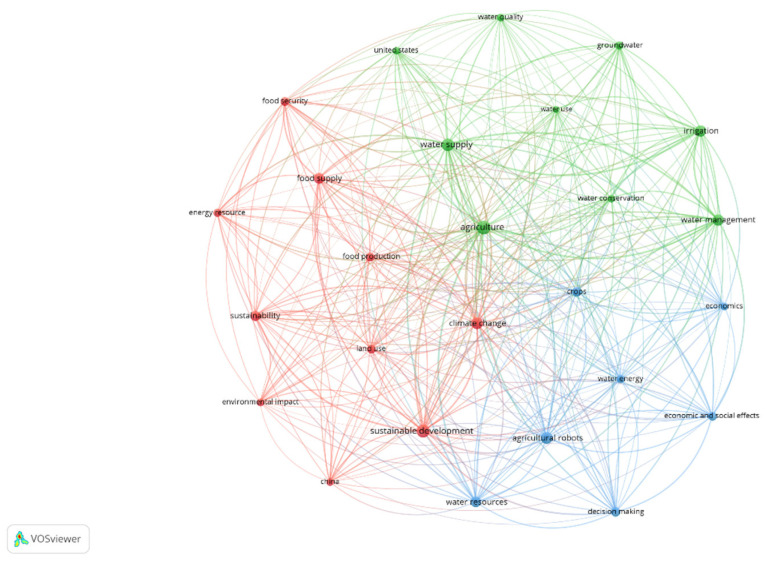
Most frequent topic words in the period 2015–2021. Source: own elaboration.

The keywords analysis serves to highlight the possible lines of research within the topic. Two different time periods were considered: 2008–2014 and 2015–2021 (Figure 6 and Figure 7). Visually [74], node size reflects the number of abstracts in which a term was present. Thus, the thickness of the lines between nodes indicates the degree of direct association between two terms, or the number of abstracts in which these terms coincide. In addition, VOSviewer groups nodes into cluster networks, a cluster being a set of tightly linked nodes, and uses colours to distinguish the different clusters.

Due to the intense evolution of the WEF Nexus theme throughout this time, significant differences can be observed between the different phases.

From 2008 to 2010, Nexus research was still an early stage of research. The Bonn Conference in 2011 pointed out that the link between water, energy, and food must be applied systematically to promote the development of the green economy [75]. At the UN Conference on Sustainable Development (Rio de Janeiro, 2012), the agricultural aspect was already introduced from the point of view of the Nexus [76]. All this made the WEF Nexus a specific object of research: Bazilian et al. [77] carried out work on quantitative simulation of the Nexus in resource security; and Hussey and Pittock [78] combined it directly with sustainable development. Continuing with the keyword analysis, three clusters are clearly visible. In the green cluster (12 nodes), the terms “energy use”, “water demand”, and “water supply” stand out, clearly referring to resource efficiency, although in a purely economic and not so ecological aspect. The blue cluster (5 nodes) is composed of “water budget”, “irrigation system”, “triticum aestitvum”, “irrigated agriculture”, and “crops”, a conceptual framework related to agricultural activity. Finally, the most used terms in the red cluster (12 nodes) are “food security”, “water productivity”, “water management”, and “irrigation”, linked to the importance of the water element of the WEF Nexus. Perhaps one aspect to note here is that environmental sustainability was still relatively unimportant compared to the Nexus components, something that would be significantly addressed in the next stage.

The latest phase began in 2015, with the Sustainable Development Goals (SDGs), which were welcomed from the outset by academics and policymakers. The SDGs are a system in which economic, environmental, and social goals interact with each other, making their link with the WEF Nexus coherent and becoming their main line of research [79,80]. In this phase, the concept of sustainable development was added to what had already been researched in the previous stages, through aspects such as sustainable governance, sustainable livelihoods, and various retrospective studies [4,81]. Figure 7 shows 26 keywords grouped into three clusters. The red cluster is made up of 10 nodes, including “sustainable development”, “climate change”, and “food supply”, in clear reference to the efficient management of resources at the environmental level. Of the 9 nodes in the green cluster, “agriculture”, “water supply”, “water management”, and “irrigation”, related to cultivation techniques. Finally, in the blue cluster, “water resources”, “agricultural robots”, “decision making”, and “crops” stand out in a conceptual framework linked to Nexus-related procedures and tools.

If we look at the most recent network map, we will notice that “agriculture” is one of the central nodes, having a multitude of connections with the nodes of the rest of the cluster. In the previous stage, this term was also one of the most mentioned. This fact can be interpreted in several ways. It may be that “agriculture” is frequently mentioned in the abstracts as a high-ranking conclusion (“agriculture is an essential activity”) or it may even be that “agriculture” is not a relevant topic at all. This aspect will be discussed again in Section 6 of this article. It is also curious to note how terms such as “sustainability”, “food security”, “water management”, and “economic and social effects”, are so far from the centre of the graph and with relatively few connections. This suggests that governance and modelling are not fully integrated in the research that has been carried out, which is also corroborated by critical reviews of the WEF Nexus literature that note that the overall research line has tended to focus on techno-economic and management aspects to the detriment of socio-political approaches [1,3].

## 5. Innovating Practices in WEF Nexus in the Agri-Food Sector

This section is focused specifically on innovative practices in the agri-food sector in the field of WEF Nexus. To do this, the additional search keyword was innovat*, obtaining 56 papers, 15 of them representing almost 80% of the total citations.

WEF Nexus innovations cover most needs to be resolved [82]. In this sense, there are resource gaps in developing countries and the Nexus research tries to repair this great problem. In works such as Bieber et al. [83], it is explained that through scenario-based holistic models, the WEF Nexus can be used sustainably; in this case, it is implemented in Ghana, extrapolating the country’s energy deficiencies and possible solutions to countries in a similar political-economic situation. The importance of sustainability in water, a scarce good in much of the world, should be highlighted and that it can be solutioned through the implementation of certain irrigation techniques [84]. At the end of the matter, it is to create as little external dependence as possible for such countries, whatever the type of resource. Van Noordwijk [85] attests to this, based on the models of “place theory” and “change theory”, which explain that sustainability methods should be based solely on the space and options of that environment, and not on external models that have little or nothing to do with the case to be studied. Theoretical management models are often based on complex mathematical formulas that, in turn, are related to chemical and biological issues; Davidson et al. [86] also address this issue, stressing that, although there are more advantages for humans than the drawbacks, within the latter we should consider the permanence in the environment of certain wastes that are the product of using efficiency techniques in obtaining natural resources, affecting environmental sustainability.

Nevertheless, the sustainability and practical implementation of the WEF Nexus are not just issues to be addressed by developing countries. The most developed countries should also cover this problem, as is evidenced by the different studies. Smidt et al. [87] have rightly summarised all this, setting an example the overexploitation that the water resources of the Great Plains (USA) have been suffering for decades, due to the lifestyle of the northern part of the Americas. Thus, it is above all a question of avoiding natural impoverishment, and the consequent economic impoverishment, of regions such as this, which are diminished by their natural resources due to a lack of real awareness of the authorities in the fight against degradation. The response to this is a series of measures, both at the small-scale management level (optimisation models to be adopted by farmers in the area and the circular economy) and on a larger scale (measures at the political and economic level). In addition to the American example, here in Europe the Association for Research and Innovation in the Mediterranean Area (PRIMA) has also taken sides in this struggle in the Mediterranean area, especially as regards the Water and Food part of the WEF Nexus [88]. Through a programme with 12 demographic, economic, and social indicators, it is intended to alleviate not only the effects of human work, but also those of droughts, floods, desertification, and soil depletion, direct effects of climate change.

As mentioned above, it is not only natural resources that are affected by lack of efficiency. Obviously, economic and business variables also suffer the negative consequences of poor management of the environmental environment. Such is the case that Kılkış and Kılkış [89], which analyse the activity of a dairy founded by a Turkish university (Ankara University). The feasibility of integrating the WEF Nexus and sustainable management within a company is studied. Beyond the economic issue, Spiegelberg et al. [90] expose not only an economic crisis at the level of the Laguna Lake region (Philippines), but a whole conflict of interests between farmers and fishermen in the area, with the consequent wear and tear of the medium that brings such discussion, while explaining how through WEF Nexus and the sociological analysis of these relations between man and nature, the exploitation of the area can be organised sustainably.

In sum, a final thematic block would itself focus on addressing the issue of tools to carry out any environmental restructuring measure in favour of environmental sustainability. Thus, the Integrated Rooftop Greenhouse (i-RTG) [91] can be firstly mentioned, an innovative farming system within its category (urban orchards), designed to improve the efficiency of cultivation in cities that use this system. Van Nooordwijk et al. [92] address the fulfilment of the Sustainable Development Goals (DGS) through environmental policies aimed at agriculture and forestry. Within the circular economy, the Biogas Condominius Method [93], based on the concept of “farm to fuel”, in which animal waste and manure are converted into electrical and thermal energy, biofuel for transport and biofertilizer. From a strictly theoretical point of view, Mayor et al. [94] conduct a study, based on the Delphi method, which attempts to predict the tendency of the WEF Nexus to analyse the safety of water and water to generate energy, over a period of 20 years from 2030 to 2050. Additionally, on a more complex technological level, McNally et al. [95] expose the use of aerospace programs to study the WEF Nexus, with the collaboration of NASA.

Finally, Table 7 sums the literature analysed in this section. The column tools indicate instruments to carry out a correct use of the WEF Nexus, within a more theorical field. Moreover, within each country, the study distinguishes between a natural environment (wild or rural environment; ultimately, not urban), or a non-natural environment (urban environment).

In addition to the bibliography extracted from Scopus, innovations extracted from reviews were studied to complement the content of the previous articles. With a number of developments ranging from natural solutions to efficiency through irrigation management and crop redistribution [96], to crop gene editing [97].

In the area of water–food linkage, Pi et al. [98] studied the role of mulching in improving soil water storage and soil properties in a maize rotation system in northwest China. Using mulching improved water storage and made the soil warmer, but decreased nitrogen content. Another example under this heading is Tsakmakis et al. [99], who demonstrated that different irrigation technologies could reduce the water footprint of cotton in a plantation in northern Greece by up to 5% using drip irrigation instead of sprinkler irrigation, and by up to 12% using deficit irrigation instead of full irrigation. Within the water–energy nexus, Abadía et al. [100] propose to assess the performance of the water distribution network through the comparison of energy audits, identifying the weaknesses of the network and taking actions accordingly to improve its efficiency. Another option would be to redistribute crops, helping to improve drought resilience through crop diversification and rotation, while optimising the spatial distribution of local cropping patterns. Through the study on 14 crops, Davis et al. [101] proved that the consumption of blue water (lakes, rivers, and aquifers) and green water (precipitation water falling on the ground without being stored) was reduced by 12.1% and 13.6%, respectively, while at the same time increasing protein and calorie production. Economically agricultural areas such as the Central Valley of California, the South-Eastern region of Australia, and the Nile Delta are also witnessing optimisation through crop distribution [96].

Advances in gene editing over the last decade have greatly improved the efficiency of breeding crop varieties with desirable traits [102] by eliminating or replacing the original genome using new technologies [103,104]. Seed companies such as DuPont have successfully employed modern CRISPR/Cas9 (Clustered Regularly Interspaced Short Palindromic Repeats) technology to improve the drought tolerance of maize [105,106]. Gene editing has also been used to improve nitrogen use in crops, reducing nitrogen fertiliser consumption and mitigating water and air pollution [107,108].

As liquid fuels are expected to remain critical over the next three decades [109,110], there is an urgent need to start replacing greenhouse gas emissions with the use of renewable fuels, which are less carbon intensive [111]. Since so-called first-generation biofuels are composed of food crops, the use of second-generation biofuels made from non-food feedstocks (agricultural residues and perennial crops) has been proposed, thus preventing them from competing for land used for food production [112].

Finally, photovoltaic panels, whose international deployment has grown exponentially due to policy incentives and cost reductions [113,114]. However, PV panels are built into the ground, diverting potentially arable land from food production [115]. Compared to the last three categories of technologies analysed, agrovoltaics has been studied the least. In 2019, Barron-Gafford et al. [116] demonstrated in vegetable crops in Arizona that agrovoltaics can decrease plant drought stress and heat stress from PV panels, with the associated increase in crop yield. According to Dupraz et al. [117], when panel density is half the optimal density (i.e., optimal for higher electricity production), durum wheat yields decrease by about 17%, but not total land yields, which increase by 35% compared to monoculture yields.

## 6. Discussion and Conclusions

The Water–Energy–Food Nexus analysis carried out provides an overview of the status and key variables of the WEFE publications in the agri-food sector. The results indicate possible future lines of research that will undoubtedly be of great use both theoretical and practically. There are several concerns to be considered:

- Variety in research methods. This bibliometric analysis was carried out by studying the information provided by Scopus, a database with extensive and diverse literature, but not only such generalist work systems should be employed. Thus, there are several specialised computer tools [118] that can be used for WEF Nexus research, such as ANEMI, CLEW, MuSIASEM, WEF Nexus Tool 2.0, and WESim. In addition, in the field of innovation stand out Albrecht et al. [119], who carried out 18 studies that will undoubtedly help the improvement of the Nexus from an analytical point of view through new procedures. Despite all this methodological variety, the WEF Nexus remains a very novel subject (the first writing dates from 1988 [1]), which makes it difficult to reduce the complexity of studying it, due to the multitude of progress that has yet to be defined. In order to reduce this diversity, the solution would then go through strengthening collaboration between disciplines, designing integrated software platforms, and inviting policy makers and stakeholders of different order to participate in this process. It is a difficult process of adaptation, due to the complexity of each system (with its individual advantages and disadvantages), as well as needing a deep understanding of the Nexus that has not yet been fully achieved, which makes it impractical today to obtain a universal method that helps to understand all the situations that may occur, both in theoretical and practical fields.
- The size or scope of the Nexus. Due to the importance of WEF Nexus in preserving equity in access to resources and sustainable development, it is necessary to know the limits that will set it. This means that the interrelationship of the elements of the system (water, energy, and food) must be studied by reference to different approaches. Thus, we must not set aside the composition of the total scheme of the Nexus, being able to increase it to add elements such as climate [120] or ecosystem [121,122]. In addition, it will be necessary to expand the horizon of traditionally regional studies to a global one, with the aim of being able to manage issues such as population growth, which would also involve interrelating scale with the economy, without forgetting that limits must always be established to allow us to avoid uncertain results. Arguably, the point of view adopted (water, energy, or food) influences the modelling of interactions, to the point of leading some authors to question whether there is really consistency in such integration [123]. Thus, water would consider energy and food as inputs, while for food the raw material would be water and energy [124]. The same situation is true for research methods. Albrecht et al. [119] reported that only a quarter of the publications on the WEF Nexus employed social science methodologies, and that the methods were generally limited to disciplinary silos. A major drawback is the lack of consistent data availability across sectors and scales: at national and transnational levels, open-access databases such as those of the Food and Agriculture Organization of the United Nations (FAO), AQUASTAT and FAOSTAT, and the United Nations Statistics Division (UNSD) are used; at smaller scales, the problem lies in commercial trust and data confidentiality [125]. Thanks to new technologies, data could be captured with better resolution in both space and time, as long as the data obtained are not excessively expensive or exclusive to institutions. Decisions are made at various levels of governance (from local, through regional and national, to global) and at various geographical scales (river basin, city, state); however, most of the models in the WEF Nexus choose a single scale of each type and do not consider inter-scalar interactions. Thus, the need for a hierarchical framework integrating all scales and perspectives arises [123]. Due to this problem, the limited action of the Nexus has come to be considered [126]. Decision support tools and models must be perceived as credible, legitimate, and salient [127].
- Lack of involvement of political authority (or, in the case of developing countries, lack of capacity to act). To implement the Nexus as efficiently as possible in a region, the development of economic strategies that foster cooperation at the inter-regional (and even inter-state) level, including trade, financial coordination, and production networks, is mandatory [128,129]. Unfortunately, due to the lack of effective monitoring and verification mechanisms, existing information is currently mostly discontinuous, scattered, and not homogeneous. Although obtaining data at the country level is possible within certain limits, it becomes complicated at lower scales (sub-national, urban, and suburban), and it is necessary to introduce quality information for a correct quantitative analysis of the Nexus. Thus, data must be consistent, comparable across scales, and available to stakeholders and the general public [40,130]. At the city level, Kennedy et al. [131] found that only three studies used models of so-called Urban Metabolism (UM), ‘the sum of the technical and socioeconomic processes that occur in cities, resulting in growth, production and energy, and elimination of waste’, to design concrete policies, and they were limited to analysing water [132] and waste processing [133,134]. Only Hendriks et al. [133] alluded to the creation of workshops as a way to seriously explore the interactions and processes governing waste management. Foran [135] went further and wrote that what is needed is a “critical social science of the Nexus”. Following published studies of cities in the United States and Europe [136,137,138], it has been observed that the management and evaluation models created are scientifically sound, but developed ‘behind closed doors’, making them unhelpful and unclear to society as a whole [139]. The solution for Van den Belt [140] is to develop such models in open and participatory spaces, creating a trust that allows acceptance by a wider group of participants. This was also the thinking of the main funding agencies in Europe, the United States, and the United Kingdom, which promoted calls for funding in 2015 through the Dear Colleague Letter (DCL), a statement of intent issued by the National Science Foundation (NSF), which called for the collaboration of research communities to unify different systems (physical, natural, and behavioural) to help the understanding of the WEF Nexus. Thus, four lines of research emerged to address in subsequent years in the first Innovations in Food, Energy, and Water Systems (INFEWS) call [125]: (I) modelling of FEW systems, (II) visualisation and decision support for implementing cyber–human–physical systems in the FEW nexus, (III) research to facilitate solutions, and (IV) education and workforce development.
- It is a relevant topic, but there are still significant research gaps, particularly in certain sectors such as agriculture. The identification of these gaps suggests opportunities to guide future WEF Nexus research. Nazmul Islam et al. [141] concluded that within the field of agriculture, Nexus research is comparatively inferior, leading to missed opportunities in water and energy savings, as well as in the consequent reduction of greenhouse gas emissions. Precision technologies will facilitate the Nexus research task by offering to collect and provide real-time data on agricultural production. However, there are several limitations, such as current data infrastructure and accessibility. For Woodard et al. [142,143], a data warehouse that is able to integrate diverse data sources, such as satellite imagery, public surveys, climate and market data, while upholding data privacy, would make the resource optimisation policies of the WEF Nexus exponentially better.
- The Sustainable Development Goals (SDGs). Following the Bonn Conference (2011), Nexus research results have increased every year [144,145], with rapid growth following the publication of the SDGs (2015), as the systematic nature of the SDGs is facilitated by Nexus thinking. Regional water, food, and energy resources can be undermined by factors such as climate change, population growth, urbanisation, and food security [42,146]. In addition, sustainable regional development must be achieved under resource constraints, which has already led to enormous challenges [147,148,149,150]. The global agricultural sector (production and supply) consumes 70% of freshwater and 30% of energy resources. With an estimated world population of 9 billion people by 2050, food supply will also need to increase by at least 60% globally [151,152]. However, the essential question remains to clarify the impact on sustainable development due to regional resource constraints: through the pressure–state–response model, the relationship between factors such as economic growth and the environment can be analysed, because it incorporates the sustainability aspect, and can serve as an exploitative framework for future research on the WEF Nexus [52,153]. Nevertheless, although studies of the Nexus have increased considerably in recent years, very few link it to the SDGs; indeed, for Boas et al. [154] the connections between many of the SDGs are weak and unstructured, and do not recognise the relationships between different sectors. The WEF Nexus is recognised in five SDGs: SDG 2 (food security), SDG 6 (clean water), SDG 7 (modern energy), SDG 13 (climate change), and SDG 15 (terrestrial ecosystems).

Since the Bonn conference and especially since the presentation of the SDGs, the importance of the Nexus has grown exponentially. The reason: the systematic and integrative aspect of the SDGs, which means that the “nexus” approach has a positive effect on their implementation. Looking at the references with the highest number of citations, the way in which the SDGs promote Nexus studies with water management will continue to be a frontier issue in the future. This article analysed research on the relationship between water and water sanitation and development in the context of regional resource constraints. Factors such as climate change, urbanisation, and population growth can lead to uncertainty in the interaction of regional water, energy, and food resources. Furthermore, within the idea of achieving sustainable regional development under resource constraints, based on the scientific theory of the planetary boundary, the SDGs related to the efficient consumption of the components of the WEF Nexus have also faced enormous challenges, also generating future lines of scientific debate.

In the Nexus studies, the key areas of research have been agricultural science, ecosystems, political governance, resource security, and sustainability. However, there are still some gaps in the study of these aspects. In terms of the object of research, existing studies almost always start from a single field or sector and consider other systems as exogenous variables; they therefore lack a correlation analysis across multiple sectors: for example, a comprehensive study on the link between agricultural water use, agricultural expansion, and deforestation. Regarding research methods, most studies use qualitative or semi-quantitative analysis (expert assessment, literature assessment, econometric modelling, objective-based decision making); however, few studies simulate the correlation between multiple systems, including research on the complex mechanisms that explain the interaction between the components of the WEF Nexus. Finally, in terms of research data, existing research is mainly based on empirical analyses and statistical data from national statistics and international organisations; few are spatio-temporal evolution data, which actually reflect the correlation process of a system.

Beyond data limitations, the main obstacle to the interpretation of the nexus studies may be due to the problem of governance scale mismatches: there remains a disconnect between the spatial scale at which decisions are made and the scale at which production and consumption are conceptualised and modelled. Decisions can be made at various governance scales (local, regional, national, and global) and geographic scales (city, county, watershed), but most of the WEF Nexus models choose a single geographic and governance scale, and do not take into account feedback between scales. This points to the need for a hierarchical framework that integrates all scales and different perspectives.

In sum, the WEF Nexus is not yet a clearly defined term within a standardised and tested framework. That is, it is still at a conceptual stage, with little empirical evidence. The agri-food Nexus has become a growing line of research over the past two decades but the lack of coordination between dimensions is the main barrier to its effective implementation.

## Figures and Tables

**Table 1:** Principal variables of Water–Energy–Food Nexus (WEF Nexus) research.

Year	Articles	Journals	Authors	Institutions	Countries	Subject Areas	Funding Sponsors	Citations	Average Citations ^1^
2008	1	1	3	3	2	1	0	17	17
2009	0	0	0	0	0	0	0	0	0
2010	0	0	0	0	0	0	0	0	0
2011	2	2	14	7	3	3	0	377	188.5
2012	1	1	4	3	3	3	1	62	62
2013	1	1	1	1	1	3	0	22	22
2014	7	7	25	13	9	10	1	198	28.29
2015	32	22	99	71	20	12	9	962	30.06
2016	40	28	153	96	20	13	34	1228	30.7
2017	37	26	126	71	20	12	22	1180	31.89
2018	64	39	160	160	45	18	88	1421	22.20
2019	80	46	160	160	39	16	95	1196	14.95
2020	83	49	160	160	44	18	116	581	7
2021	115	56	160	160	44	16	112	233	2.03

^1^ Number of citations divided by the number of articles. Source: own elaboration.

**Table 2:** Main journals in the WEF Nexus.

Journals	Articles	SJR ^1^	Sample H Index (Global H Index ^1^)	Countries	Citations	Average Citations ^2^	First Article	Last Article	Founding Year
Journal of Cleaner Production	30	1.937 (Q1)	13 (200)	UK	454	15.13	2015	2021	1993
Science of the Total Environment	27	1.795 (Q1)	15 (244)	The Netherlands	656	24.3	2016	2021	1972
Water (Switzerland)	24	0.718 (Q1)	6 (55)	Switzerland	195	8.13	2016	2021	2009
Sustainability (Switzerland)	22	0.612 (Q1)	7 (85)	Switzerland	107	4.86	2016	2021	2009
Resources, Conservation and Recycling	19	2.468 (Q1)	5 (130)	The Netherlands	89	4.68	2017	2021	1988
Environmental Research Letters	15	2.370 (Q1)	10 (124)	UK	484	32.27	2014	2021	2006
Environmental Science and Policy	11	1.716 (Q1)	8 (115)	The Netherlands	318	28.91	2016	2020	1998
Applied Energy	10	3.035 (Q1)	8 (212)	UK	375	37.5	2016	2021	1975
Frontiers in Environmental Science	9	1.225 (Q1)	7 (37)	Switzerland	118	13.11	2017	2019	2013
Sustainable Production and Consumption	9	1.019 (Q1)	6 (26)	The Netherlands	244	27.11	2015	2021	2015

^1^ SCImago Journal Rank 2020; ^2^ number of citations divided by the number of articles. UK: United Kingdom, USA: United States. Source: own elaboration.

**Table 3:** Top-10 authors in WEF Nexus.

Authors	Average Citations ^1^	Citations	Articles	H Index ^2^	Countries	Affiliation	First Article	Last Article
D’Odorico, Paolo	52.2	261	5	5	USA	Department of Environmental Science, Policy and Management, and University of California	2016	2019
Fu, Qiang.	41	164	4	3	China	Northeast Agricultural University	2019	2021
Campana, Pietro Elia	36.25	145	4	4	Sweden	Mälardalens högskola	2015	2019
Liu, Dong	34	170	5	4	China	Northeast Agricultural University	2019	2021
Li, Mo W.	34	170	5	4	China	Northeast Agricultural University	2019	2021
Mohtar, Rabi H.	17	85	5	4	Lebanon	American University of Beirut	2018	2020
Daher, Bassel T.	15	75	5	4	USA	Texas A&M University	2019	2021
Al-Ansari, Tareq	14.42	173	12	7	Qatar	Hamad Bin Khalifa University	2015	2021
Taniguchi, Makoto	13.2	66	5	5	Japan	National Institutes for the Humanities, Research Institute for Humanity and Nature	2017	2020
Govindan, Rajesh	7.2	36	5	3	Qatar	Hamad Bin Khalifa University, College of Science and Engineering	2020	2021

^1^ Number of citations divided by the number of articles; ^2^ sample articles. Source: own elaboration.

**Table 4:** Most relevant institutions in WEF Nexus.

Institutions	Articles	H Index ^1^	Countries	Citations	Average Citations ^2^	Founding Year
Texas A&M University	17	10	USA	349	20.53	1871
Beijing Normal University	15	8	China	307	20.47	1902
Chinese Academy of Sciences	14	9	China	167	11.93	1949
Wageningen University and Research Centre	13	7	Netherlands	203	15.62	1876
Hamad Bin Khalifa University	11	6	Qatar	75	6.82	2010
Hamad Bin Khalifa University, College of Science and Engineering	11	6	Qatar	75	6.82	2010
The Ohio State University	10	5	USA	128	12.8	1870
The Royal Institute of Technology KTH	9	6	Sweden	241	26.78	1827
National Taiwan University	9	6	Taiwan	122	13.56	1928
China Agricultural University	9	4	China	126	14	1905

^1^ Sample articles; ^2^ number of citations divided by the number of articles. Source: own elaboration.

**Table 5:** Most active countries in WEF Nexus.

Countries	Articles	Average Per Capita Articles ^1^	H Index ^2^	Citations	Average Citations ^3^	First Article	Last Article
USA	178	0.5402	30	3333	18.72	2011	2021
China	78	0.0556	15	890	11.41	2016	2021
UK	59	0.8777	9	1312	22.24	2013	2021
Germany	34	0.4085	9	478	14.06	2014	2021
Italy	34	0.5709	9	598	17.59	2015	2021
The Netherlands	27	1.5482	7	374	13.85	2016	2021
Spain	27	0.5702	7	448	16.59	2014	2021
India	22	0.0159	9	340	15.45	2011	2021
Canada	21	0.5525	7	290	13.81	2015	2021
Brazil	20	0.0941	4	95	4.75	2015	2021

^1^ Number of articles per million inhabitants; ^2^ sample articles; ^3^ number of citations divided by the number of articles. Source: own elaboration.

**Table 6:** Main funding sponsors in WEF Nexus.

Funding Sponsors	Articles	H Index ^1^	Countries	Citations	Average Citations ^2^	First Article	Last Article	Founding Year
National Science Foundation	50	16	USA	842	16.84	2015	2021	1950
National Natural Science Foundation of China	49	13	China	687	14.02	2017	2021	1986
European Commission	15	8	EU	198	13.2	2018	2021	1958
Horizon 2020 Framework Programme	15	8	EU	174	11.6	2018	2021	2014
Chinese Academy of Sciences	14	6	China	149	10.64	2018	2021	1949
Natural Environment Research Council	11	8	UK	253	23	2015	2021	1965
Coordenação de Aperfeiçoamento de Pessoal de Nível Superior	10	4	Brazil	31	3.1	2016	2021	1951
Conselho Nacional de Desenvolvimento Científico e Tecnológico	9	3	Brazil	21	2.33	2020	2021	1951
Consortium of International Agricultural Research Centers	9	6	France	207	23	2012	2020	1971
National Key Research and Development Program of China	9	3	China	23	2.56	2020	2021	2016

^1^ Sample articles; ^2^ number of citations divided by the number of articles. Source: own elaboration.

**Table 7:** Most cited articles about innovation in WEF Nexus.

				Topic			
Articles and Reviews	Author/s	Years	Journals	Tools	Developed Countries	Developing Countries	Citations
Article: Sustainable planning of the energy–water–food nexus using decision making tools	Bieber, N., Ker, J.H., Wang, X., Triantafyllidis, C., van Dam, K.H., Koppelaar, R.H.E.M., Shah, N.	2018	Energy Policy	To ensure that, through tools and proposals, in agricultural and urban areas, affecting the least developed countries, WEF Nexus is used sustainably.	-	Natural environment	93
Article: Complex water management in modern agriculture: Trends in the water–energy–food nexus over the High Plains Aquifer	Smidt, S.J., Haacker, E.M.K., Kendall, A.D., Deines, J.M., Pei, L., Cotterman, K.A., Li, H., Liu, X., Basso, B., Hyndman, D.W.	2016	Science of the Total Environment	-	Natural environment	-	61
Article: Environmental assessment of an integrated rooftop greenhouse for food production in cities	Sanjuan-Delmás, D., Llorach-Massana, P., Nadal, A., Ercilla-Montserrat, M., Muñoz, P., Montero, J.I., Josa, A., Gabarrell, X., Rieradevall, J.	2018	Journal of Cleaner Production	Integrated rooftop greenhouse (i-RTG) (innovative crop system within its category (urban gardens)), which contributes to sustainability.	-	-	58
Article: Closing the yield gap while ensuring water sustainability	Rosa, L., Rulli, M.C., Davis, K.F., Chiarelli, D.D., Passera, C., D’Odorico, P.	2018	Environmental Research Letters	Sustainability in the field through new irrigation techniques (also incidence in less developed countries).	-	Natural environment	54
Review: SDG synergy between agriculture and forestry in the food, energy, water and income nexus: reinventing agroforestry?	van Noordwijk, M., Duguma, L.A., Dewi, S., Leimona, B., Catacutan, D.C., Lusiana, B., Öborn, I., Hairiah, K., Minang, P.A.	2018	Current Opinion in Environmental Sustainability	Agriculture and forestry management for environmental sustainability.	-	-	43
Article: Linking the water–energy–food nexus and sustainable development indicators for the Mediterranean region	Saladini, F., Betti, G., Ferragina, E., Bouraoui, F., Cupertino, S., Canitano, G., Gigliotti, M., Autino, A., Pulselli, F.M., Riccaboni, A., Bidoglio, G., Bastianoni, S.	2018	Ecological Indicators	-	Natural and non-natural environment	Natural and non-natural environment	42
Article: Integrated circular economy and education model to address aspects of an energy–water–food nexus in a dairy facility and local contexts	Kılkış, Ş., Kılkış, B.	2017	Journal of Cleaner Production	-	-	Non-natural environment	38
Article: Integrated natural resource management as pathway to poverty reduction: Innovating practices, institutions and policies	van Noordwijk, M.	2019	Agricultural Systems	-	Natural environment	Natural environment	33
Article: Nutrients in the nexus	Davidson, E.A., Nifong, R.L, Ferguson, R.B., Palm, C., Osmond, D.L., Baron, J.S.	2016	Journal of Environmental Studies and Sciences	Nitrogen management; reduce in developed and less developed countries, and increase in underdeveloped countries.	-	Natural environment	22
Article: Unfolding livelihood aspects of the Water–Energy–Food Nexus in the Dampalit Watershed, Philippines	Spiegelberg, M., Baltazar, D.E., Sarigumba, M.P.E., Orencio, P.M., Hoshino, S., Hashimoto, S., Taniguchi, M., Endo, A.	2017	Journal of Hydrology	-	-	Non-natural environment	20
Article: Nexus narratives and resource insecurities in the Mekong Region	Lebel, L., Lebel, B.	2018	Environmental Science and Policy	-	-	Natural environment	19
Article: Assessment of Collective Production of Biomethane from Livestock Waste for Urban Transportation Mobility in Brazil and the United States	Pasqual, J.C., Bollmann, H.A., Scott, C.A., Edwiges, T., Baptista, T.C.	2018	Energies	Biogas Condominius: Based on the concept of “farm to fuel”, animal waste and manure are converted into electrical and thermal energy, biofuel for transport and biofuel.	-	-	12
Article: Implications of non-sustainable agricultural water policies for the water-food nexus in large-scale irrigation systems: A remote sensing approach	Al Zayed, I.S., Elagib, N.A.	2017	Advances in Water Resources	-	-	Natural environment	12
Article: An expert outlook on water security and water for energy trends to 2030–2050	Mayor, B., Casado, R.R., Landeta, J., López-Gunn, E., Villarroya, F.	2016	Water Policy	Study, based on the Delphi method (statistics), to anticipate the tendency of the WEF Nexus to analyse the safety of water and water to generate energy. Some sections would be interesting for the theoretical WEF Nexus section.	-	-	11
Review: Hydrologic and agricultural Earth observations and modelling for the water–food nexus	McNally, A., McCartney, S., Ruane, A.C., Mladenova, I.E., Whitcraft, A.K., Becker-Reshef, I, Bolten, J.D., Peters-Lidard, C.D., Rosenzweig, C., Uz, S.S.	2019	Frontiers in Environmental Science	Use of aerospace programs to study the WEF Nexus (ERTS, AVHRR, NDVI, GEOS-5).	-	-	7

Source: own elaboration.

## Abbreviations

ANEMI	An ancient Greek term for the four winds, heralds of the four seasons.
CLEW	Climate, Land, Energy and Water.
CRISPR/Cas9	Clustered Regularly Interspaced Short Palindromic Repeats.
CSV format	Comma-Separated Values format.
DCL	Dear College Letter.
EU	European Union.
FAO	Food and Agriculture Organization of the United Nations.
i-RTG	Integrated Rooftop Greenhouse.
IF	Impact Factor.
INFEWS	Innovations in Food, Energy and Water Systems.
KTH	Royal Institute of Technology: Kungliga Tekniska högskolan (Swedish).
LAC	Latin America and Caribbean.
MuSIASEM	Multi-Scale Integrated Analysis of Societal and Ecosystem Metabolism.
NASA	National Aeronautics and Space Administration.
NSF	National Science Foundation.
PRIMA	Association for Research and Innovation in the Mediterranean Area.
PUBYEAR	Publication year.
PV panels	Photovoltaic panels.
Q1	Quartile 1.
REDD+ programme	Reducing Emissions from Deforestation and Forest Degradation programme.
RIS format	Research Information Systems format.
SJR	SCIMago Journal Rank.
SciMAT	Soft Computing and Intelligent Information Systems Research group.
SDGs	Sustainable Development Goals.
Texas A&M University	Texas Agricultural and Mechanical University.
UK	United Kingdom.
UM	Urban Metabolism.
UN	United Nations.
USA	United States of America.
UNSD	United Nations Statistics Division.
WEF Nexus	Water–Energy–Food Nexus.
WEFE	Water-–Energy–Food–Ecosystems.
WESim	The Water-Energy Simulator.
